# Abdominoscrotal Hydrocele: An Uncommon Cause of Abdominoscrotal Cystic Swelling

**DOI:** 10.1155/2021/6655127

**Published:** 2021-07-09

**Authors:** Manish Swarnkar, Pathan Tanveer Khan

**Affiliations:** ^1^Department of Surgery, LN Medical College, Madhya Pradesh, 462042, Bhopal, India; ^2^Department of General Surgery, JNMC, Maharashtra, 442001, Wardha, India

## Abstract

Abdominoscrotal hydrocele (ASH) consists of fluid-filled intercommunicating inguinoscrotal and abdominal sac with a characteristic hourglass-like picture on CECT, which usually affects single testis and a rare cause of abdominoscrotal cystic swelling. The precise etiology of ASH is not known. Ultrasonography is the initial diagnostic modality of choice as it demonstrates the intercommunication between the two sacs and also identifies any abnormality of the testis and genitourinary tract. We are reporting a case of a 27-year-old patient presented in the General Surgery OPD of Acharya Vinoba Bhave Hospital in 2019 with bilateral scrotal and abdominal swelling. On examination, cross fluctuation was positive between left hydrocele and abdominal swelling, raising suspicion of ASH, which was confirmed on CECT. The patient underwent excision of sac through left inguinoscrotal approach and an uneventful postoperative course.

## 1. Introduction

Abdominoscrotal hydrocele is a rare clinical entity seen only in 0.18%-3.1% of hydrocele cases [1, 2]. ASH is a dumbbell or hourglass-shaped hydrocele that extends from the scrotum to the abdominal cavity extraperitonealy through the inguinal canal. ASH is usually unilateral, but few bilateral cases have been described in literature. Here, we present a case of left-sided ASH with review of literature.

## 2. Case Report

**Figure 1:**
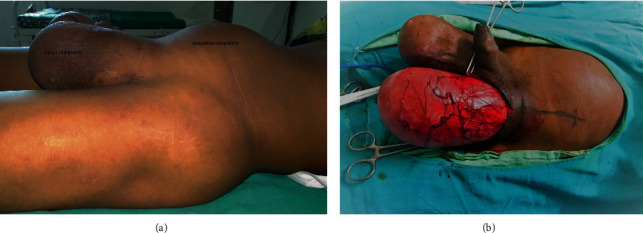
(a) Photograph showing abdominal and scrotal components of abdominoscrotal hydrocele. (b) Excision of sac through inguinoscrotal incision.

**Figure 2:**
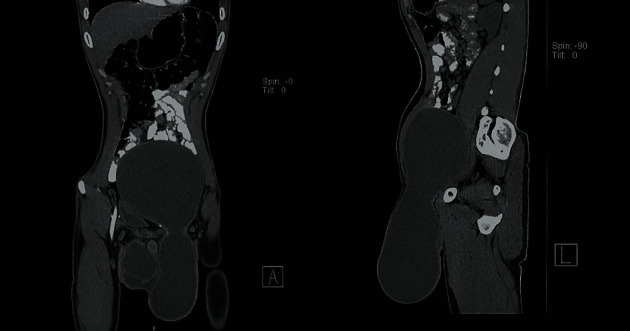
CECT showing hourglass appearance of ASH with intercommunicating scrotal and abdominal sac.

A 27-year-old male patient presented in the General Surgery OPD of Acharya Vinoba Bhave Hospital in 2019 with bilateral scrotal and lower abdominal swelling (Figure 1(a)), which was insidious in onset with dull aching dragging pain. There was no previous history of trauma or fever. On physical examination, bilateral hydrocele was present, and both swellings were nonreducible, transluminant, and fluctuant. Abdominal swelling was extending up to the umbilicus, and cross fluctuation was present with left side hydrocele, raising the possibility of left-sided ASH. The CECT abdomen revealed a large intercommunicating homogeneous hourglass-shaped fluid collection involving the lower abdomen and left scrotum (Figure 2), confirming the diagnosis. The patient underwent uneventful, complete excision of the sac through the left inguinoscrotal approach (Figure 1(b)).

## 3. Discussion

Since the first description by Dupuytren in 1834 when it was called hydrocele en bisac, many titles have been used until Bickle in 1919 suggested the abdominoscrotal hydrocele as a proper descriptive term [1, 3]. ASH is most commonly seen in the pediatric age group of less than five years and second and third decade in adults. It is a congenital anomaly of processus vaginalis which starts as an inguinoscrotal hydrocele and gradually extends into the abdomen forming a two-compartment intercommunicating hydrocele [1]. Free intercommunication is a cardinal feature giving rise to characteristic clinical and radiological features [1]. The concepts of ASH etiopathogenesis are basically based on intraoperative findings. The three most commonly proposed theories are “(1) cephalad extension of a simple hydrocele, (2) high obliteration of processus vaginalis (PPV), and (3) PPV acting as a one-way valve with cephalad extension of hydrocele sac.” However, the most widely accepted theory is Dupuytren's original theory of high intracystic pressure in the scrotal component that leads to cephalad extension through the musculofascial inguinal canal and formation of the abdominal sac [4]. The natural history of ASH varies in pediatrics and adults; in children, it is a rapidly evolving observable lesion with the possibility of spontaneous resolution, but in adults, it is a long-standing, nonresolving progressive cystic lesion [1]. “Springing back ball sign” firstly suggested by Wlochynski et al. in which compression of the scrotum makes the abdominal component more prominent and once pressure is released scrotum regains its size is more characteristic than the cross fluctuation test [1, 5]. Testicular ectopia or cryptorchidism is the most common congenital anomalies associated with ASH [1].

USG is the initial modality of investigation in which ASH appears as an anechoic homogeneous lesion and intercommunication can be delineated on graded compression during USG [1, 6]. The CECT abdomen is required to prove definitive intercommunication and in complex cases. Magnetic resonance imaging is required to detect vascular complication like deep vein thrombosis and for suspicion of malignant transformation [1, 6].

ASH should be differentiated from hernia, chord lymphangioma, spermatocele, cystic abdominal mass, and ascites. Long-standing ASH leads to pressure-related complication like hydroureteronephrosis, deep vein thrombosis, leg edema, testicular dysraphism, and impaired spermatogenesis and rarely malignant transformation [4, 7, 8].

Malignancy with ASH seen only in 0.86% of cases, because of low incidence and serial pathological examination of specimens to rule out malignancy, could not provide any evidence that ASH is responsible for malignant transformation [1].

Surgical excision of the sac is the cornerstone of treatment in adult patients because of the unlikelihood of spontaneous resolution, to avoid pressure-related complications [4]. Different surgical techniques like scrotal, inguinal, inguinoscrotal, and laparoscopic-assisted scrotal approaches have been described in literature. The inguinoscrotal approach provides excellent exposure, and further dissection can be facilitated by decompression of sac or cutting of deep inguinal ring which helps in identification and mobilization of vas deferens and spermatic vessels for orchidopexy. In difficult cases, paramedian laparotomy may be required to dissect the sac away from the urinary bladder and other retroperitoneal structure [4]. Conservative management has been described in asymptomatic, uncomplicated pediatric ASH where spontaneous resolution is possible, or the patient is a poor candidate for surgery [1, 9].

## 4. Conclusion

ASH is a rare cause of abdominoscrotal swelling, which has different etiological hypotheses and multiple clinicopathological variants. Clinical examination and USG are initial modalities sufficient to make diagnoses, but CECT is required to prove intercommunication and other associated complexities. Despite available minimal invasive techniques for treatment, complete excision of the sac via inguinoscrotal incision remains the standard approach.
